# Root Morphogenesis of *Arabidopsis thaliana* Tuned by Plant Growth-Promoting *Streptomyces* Isolated From Root-Associated Soil of *Artemisia annua*

**DOI:** 10.3389/fpls.2021.802737

**Published:** 2022-01-10

**Authors:** Wenbo Fu, Yanshuo Pan, Yuhua Shi, Jieyin Chen, Daozhi Gong, Yuzhong Li, Guangfei Hao, Dongfei Han

**Affiliations:** ^1^School of Life Sciences and Food Engineering, Hebei University of Engineering, Handan, China; ^2^Institute of Environment and Sustainable Development in Agriculture, Chinese Academy of Agricultural Sciences, Beijing, China; ^3^College of Natural Resources and Environment, Northwest Agriculture and Forestry University, Yangling, China; ^4^Key Laboratory of Beijing for Identification and Safety Evaluation of Chinese Medicine, Institute of Chinese Material Medica, China Academy of Chinese Medical Sciences, Beijing, China; ^5^State Key Laboratory for Biology of Plant Diseases and Insect Pests, Institute of Plant Protection, Chinese Academy of Agricultural Sciences, Beijing, China

**Keywords:** *Streptomyces lincolnensis*, PGPR, indole-3-acetic acid (IAA), root morphogenesis, endophytic microbes

## Abstract

In this study, the capacity to tune root morphogenesis by a plant growth-promoting rhizobacterium, *Streptomyces lincolnensis* L4, was investigated from various aspects including microbial physiology, root development, and root endophytic microbial community. Strain L4 was isolated from the root-associated soil of 7-year plantation of *Artemisia annua*. Aiming at revealing the promotion mechanism of *Streptomyces* on root growth and development, this study first evaluated the growth promotion characters of *S*. *lincolnensis* L4, followed by investigation in the effect of L4 inoculation on root morphology, endophytic microbiota of root system, and expression of genes involved in root development in *Arabidopsis thaliana*. *Streptomyces lincolnensis* L4 is able to hydrolyze organic and inorganic phosphorus, fix nitrogen, and produce IAA, ACC deaminase, and siderophore, which shaped specific structure of endophytic bacterial community with dominant *Streptomyces* in roots and promoted the development of roots. From the observation of root development characteristics, root length, root diameter, and the number of root hairs were increased by inoculation of strain L4, which were verified by the differential expression of root development-related genes in *A*. *thaliana*. Genomic traits of *S*. *lincolnensis* L4 which further revealed its capacity for plant growth promotion in which genes involved in phosphorus solubilization, ACC deamination, iron transportation, and IAA production were identified. This root growth-promoting strain has the potential to develop green method for regulating plant development. These findings provide us ecological knowledge of microenvironment around root system and a new approach for regulating root development.

## Introduction

Rhizosphere is the soil zone surrounding plant root, which derive but differ from bulk soil (Hartmann et al., 2007). The interaction between root and rhizosphere soil shaped specific physical and chemical character of rhizosphere soil, and also the structure and function of microbial community (Fan et al., 2020). As a consequence, rhizosphere microbes also take effect on the growth and development of host plant. It has been intensively reported that microbes associated with root systems are diverse and usually demonstrate beneficial activity to host plant by stimulating plant growth, reducing pathogenesis, and alleviating abiotic stresses, which are assigned as plant growth-promoting rhizobacteria (PGPR). These PGPR are crucial component of phytomicrobiome that is associated with physiological regulation in plants (Smith et al., 2015). The function of rhizosphere microbes in plant phenotype regulation has important implications for plant phenology in various climates and for increasing agricultural production (Singh et al., 2010).

During the interaction between rhizosphere microbes and host plant, auxins synthesized by rhizosphere microorganisms play a critical role (Reyes, 2006; Li et al., 2018; Treesubsuntorn et al., 2018). The function of auxins from microorganisms as phytohormone raised the intriguing possibility for regulating plant growth and development by utilizing rhizosphere microbes or synthetic microbial consortium (Chen et al., 2017). According to the study of isolated PGPR and their physiological trait, the function of PGPR has been reported as inhibiting phytopathogens (Rai and Nabti, 2017), improving plant nutrient supply, producing phytohormones, and modifying physicochemical properties of soil (Khan et al., 2020). On the other hand, these PGPR strains are also able to enhance the disease resistance of plants through changing soil microbial community (Bharti et al., 2015; Wang et al., 2020).

*Streptomyces* spp. are ubiquitous actinobateria in soils, rhizosphere, and endophytic microbiota. At present, the applications of *Streptomyces* in agriculture are mainly as biocontrol agents (Yuan and Crawford, 1995). However, for application as a PGPR, the promotion mechanism of *Streptomyces* still needs further investigation. It has been reported in some studies that *Streptomyces* is able to promote plant growth by the producing 1-aminocyclopropane-1-carboxylate deaminase (ACC), indole-3-acetic acid (IAA), and siderophores to cope with abiotic stress (El-Tarabily, 2008; Rungin et al., 2012). However, little is known about the role of *Streptomyces* in host root development (Wu et al., 2019).

In our previous study, the increased abundance of actinobacteria was observed in root-associated soil with 7-year plantation of *Artemisia annua* (Shi et al., 2021). It is the only raw material for the production of artemisinin, which is the most effective drug to treat malaria so far. The enrichment of actinobacteria implied that they may have connection with the growth of *A*. *annua*. Thus, in this study, the actinobacterial strains that harbor plant growth-promoting character were isolated from the root-associated soil with 7-year plantation of *A*. *annua*. Specifically, the function in plant growth promotion and root development of actinobacterial isolate was further investigated.

## Materials and Methods

### Soil Sampling and Bacteria Isolation

The root-associated soils of *A*. *annua* YQ2 were sampled from the field with 7-year plantation history at Rong’an in China. Due to the annual plantation of *A*. *annua*, the soils that have been plowed every year were actually the mixture of 7-year accumulated rhizosphere soil. Five rhizosphere sample replicates were collected by shaking off the soil which tightly adhered to root. The replicate that showed the highest enrichment of actinobacteria in our previous study (Shi et al., 2021) was selected for bacterial isolation in this study.

For the isolation of actinobacteria, one gram of homogenized rhizosphere soil was inoculated to LB liquid medium and incubated in 28°C with 150 rpm rotary shaking for 24 h. The enriched soil microbial culture was diluted for 10, 100 and 1,000 times gradient. These diluted liquid cultures (200 μL for each sample) were spread on actinobacteria selecting Gause’s agar medium (20 g L^–1^ soluble starch, 1 g^–1^ KNO_3_, 0.5 g L^–1^ K_2_HPO_4_, 0.5 g L^–1^ MgSO_4_ ⋅ 7H_2_O, 0.5 g L^–1^ NaCl, 0.01 g L^–1^ FeSO_4_ ⋅ 7H_2_O, 20 g L^–1^ agar, 1 L of distilled water, pH = 7.4–7.6) and incubated in 28°C for 5 days.

### Identification of Actinomyces

The colonies from selective medium were picked and cultured rotationally in 5 ml of liquid LB medium for 48 h at 30°C. Genomic DNA of each culture was isolated by bacterial DNA extraction kit (OMEGA Bacterial DNA Kit). The purified DNA was resuspended in TE buffer and stored at -20°C for further analysis.

For identifying taxonomy of isolated strains, the sequence of 16S rRNA gene was amplified using the bacterial-specific primers, 27F (5′-AGAGTTTGATCCTGGCTCAG-3′) and 1492R (5′-GGTTACCTTGTTACGACTT-3′). Polymerase chain reaction (PCR) amplifications were performed with 2 × Taq PCR Mix (TIANGEN BIOTECH, China), 1.0 μM of each primer, genomic DNA as template and ddH_2_O. Three independent PCR amplifications for each isolate were performed for 30 cycles with denaturation at 94°C (60 s), annealing at 55°C (30 s), and extension at 72°C (60 s), followed by a final extension at 72°C (10 min). The resulted PCR products were sequenced with platform (SinoGenoMax, China). The taxonomy of each isolate was confirmed by the alignment of these amplified sequences with NR database in GenBank with BLAST and the comparison with nucleotide sequence homology of 16S rRNA gene for bacteria. Highly homologous sequences were aligned, and neighbor joining trees were generated using Molecular Evolutionary Genetics Analysis X.

### Microbial Physiological Assay of Bacterial Isolates

#### Phosphate Solubilization

Phosphate solubilization abilities of all isolates were screened according to the method of Surange et al. (1997). The tests were carried out in triplicates. The presence of lysis circle around the bacterial colony was observed as indicator of phosphate solubilization. The diameters of the lysis circle and colony were measured digitally. The ability of phosphate solubilization was represented by phosphorus solubilizing index (SI) based on the definition: SI = (diameter of halo zone + colony diameter)/colony diameter.

#### Nitrogen Fixation

The capability of autotrophic nitrogen fixation in isolated strains was characterized by detecting their growth in nitrogen-free medium. The isolated bacteria were inoculated in nitrogen-free medium (5.0 g mannitol or glucose L^–1^, 0.2 g KH_2_PO_4_ L^–1^, 0.2 g MgSO_4_ ⋅ 7H_2_O L^–1^, 0.2 g NaCl L^–1^, 0.2 g CaSO_4_ ⋅ 2H_2_O L^–1^, 5 g CaCO_3_ L^–1^, 15 g agar L^–1^, 1 L of distilled water, pH = 7.0–7.2). These cultures were incubated on the nitrogen-free medium at 30°C for 5 days. The formation of bacterial colony was defined as the ability of nitrogen fixation.

#### Siderophore Production

According to the method of Schwyn and Nelands (1987), the blue agar chrome azurol S (CAS) medium was used to characterize the siderophore production potential of all bacterial isolates (Alexander and Zuberer, 1991). Quantification of siderophore produced from each isolated strain was performed in liquid medium supplemented with CAS. These isolates with a yellowish orange halo were further inoculated into liquid MKB medium and incubated at 30°C for 3 days. Then, cell-free supernatants from the culture were collected by centrifugation (10,000 × *g* for 5 min), and 3 mL of the bacterial supernatant was added into the same amount of CAS solution, followed by dark incubation for 1 h. The uninoculated supernatant was used as blank control for comparison and calculation. All treatments were performed in triplicates. Absorbance of the isolates was measured at a wave length of 630 nm using spectrophotometer. The ratio, A/Ar (OD_630_ of treatments/OD_630_ of blank control), represented the capacity of producing siderophores.

#### Indole-3-Acetic Acid Production

The IAA production capacities of the strains were determined according to the method of Patten and Glick (2002). The IAA concentration was calculated from a calibration curve of IAA standard solution ranging from 0 to 20 mg/L (Supplementary Figure 1).

#### 1-Aminocyclopropane-1-Carboxylate Deaminase Activity Assay

For investigating the activity of ACC deaminase, each isolated strain was inoculated on ADF solid medium and cultured at 30°C for 7 days. If the strain could grow, it was transferred to the liquid medium with ACC as the sole nitrogen source (Jaemsaeng et al., 2018). The strain that kept growing after three generations was defined as ACC deaminase active strain.

### Genome Sequencing and Annotation

#### Genome Sequencing

Genomic DNA was extracted with the SDS method (Lim et al., 2016). Sequencing libraries were generated using NEBNext^®^ Ultra™ DNA Library Prep Kit for Illumina (NEB, United States) referring to the manufacturer’s recommendations. Purified libraries (AMPure XP system) were analyzed for size distribution by Agilent 2100 Bioanalyzer and quantified using real-time PCR. Library sequencing was performed with Illumina HiSeq/NovaSeq PE150 platform at the Beijing Allwegene Technology Co., Ltd. Quality filtered paired reads were assembled by the SPAdes (v3.13.0) (Bankevich et al., 2012) software into a number of scaffolds. Finally, scaffolds with larger than 500 bp were selected for subsequent analysis.

#### Genome Annotation

The genome was annotated using Kyoto Encyclopedia of Genes and Genomes (KEGG; Kanehisa et al., 2021). Genome component including the coding gene, repetitive sequences, noncoding RNA, protein signal peptide, secretory protein, pseudogene, prophage, genomics islands, and clustered regularly interspaced short palindromic repeat (CRISPR) sequences were comprehensively predicted. Coding genes involved in plant growth promoting were screened manually.

### Measurement of Plant Growth and Root Development

The gnotobiotic root elongation assay was applied for assessing the effect of bacterial isolates on the growth of *Arabidopsis thaliana* Col-0 seedlings. Col-0 seeds were surface sterilized before cultivation. The seeds (approximately 0.2 g per treatment) were washed in 70% ethanol for 1 min followed by washing with 1% sodium hypochlorite for 8 min. These sterilized seeds were further rinsed with sterile distilled water at least five times for removing sterilizing reagent. After sterilization, 15 seeds were placed in half-cut MS agar medium with sterilized scalpel and tweezer. The supernatant of 100 μL bacterial solution that was precultured in liquid LB (after 3 days of activation in LB liquid medium, stand for about 2 h, wait for the cell to precipitate naturally, and measure the number of supernatant spores by coating method, 400–600 mL^–1^) was inoculated on the surface of seeds (liquid LB and sterile water were used as control). Each plate was placed vertically in a 28°C greenhouse, with 12-h light/dark cycles (18 μmol m^–2^ s^–1^). The primary root lengths were measured after 21 days.

### Bacterial Communities Associated With the Roots of *Arabidopsis thaliana*

#### Root Sampling

The *Arabidopsis* plants in plate were sampled and washed with sterile water to clean the medium residues. The roots were collected for further endophytic microbial community analysis. All treatments were conducted in triplicates.

#### DNA Extraction, Amplification, and Sequencing

Total genomic DNA was extracted using DNA isolation kit (MoBio Laboratories, Carlsbad, CA, United States) following the manual. Purity and quality of the genomic DNA were checked on 1% agarose gels and NanoDrop spectrophotometer (Thermo Scientific). The bacterial 16S rRNA gene (V3–V4) was amplified by primer pairs 338F (5’-ACTCCTACGGGAGGCAGCAG-3’)/806R (5’-GGACTACHVGGGTWTCTAAT-3’) (Xu et al., 2016). The PCR was carried out on a Mastercycler Gradient (Eppendorf, Germany) (Yan et al., 2022). Amplicon sequencing was performed on Miseq platform at Allwegene, Beijing. Index and barcode sequences were removed from resulted reads, and quality was controlled using Illumina Analysis Pipeline version 2.6.

All raw data sequenced were deposited in the NCBI Sequence Read Archive (SRA) with the accession number PRJNA761252.

#### Sequence Analysis and Annotation

The raw data were first screened by removing the sequences shorter than 230 bp or with a low-quality score (≤20). These filtered sequences were separated using the sample-specific barcode sequences. Qualified reads were clustered into operational taxonomic units (OTUs) at a similarity level of 97% (Edgar, 2013) using Uparse algorithm of Vsearch (v2.7.1) software.

QIIME (v1.8.0) was used to generate rarefaction curves and to calculate the richness and diversity indices based on the OTU information. To compare the number of communities in samples of different components and structures, stacked bar chart was built (Jami et al., 2013). Based on the results of taxonomic annotation and relative abundance, R (v3.6.0) software was used for bar-plot diagram analysis.

### Quantitative PCR of Root Development-Related Genes in *Arabidopsis thaliana*

The treated and control *A*. *thaliana* was sampled after 21 days of incubation and washed with sterile water to clean off the attached medium. The root samples were cut and grinded directly in the Total RNA Extractor (Sangon Biotech) with a proportion as 100 mg samples per 1mL solution. Total RNA was extracted according to the instructions (Sangon Biotech). Qualified RNA was reversely transcribed into cDNA by reverse transcription kit, and then the genes controlling root length and root hair were quantified.

The cDNA template was diluted 10–20 times, according to the instructions of SYBR Premix Ex Taq™ (perfect real time) (Takara) kit, and PCR (CFX96TOUCH) experiments were performed. PCR amplifications were performed with 2 × Taq SYBR Green qPCR Premix (Takara, Japan), 1.0 μM of each primer, genomic DNA as template and ddH_2_O. Three independent PCR amplifications for each isolate were performed for 45 cycles with denaturation at 94°C (30 s), annealing at 57°C (30 s), and extension at 72°C (30 s). Each experiment was performed in triplicate. The primer sequence for quantitative PCR was shown in Supplementary Table 1. Gene encoding 18S rRNA was used as internal reference for calibration and comparison.

## Results

### *Streptomyces* spp. Isolated From Root-Associated Soil in *Artemisia annua*

**FIGURE 1:**
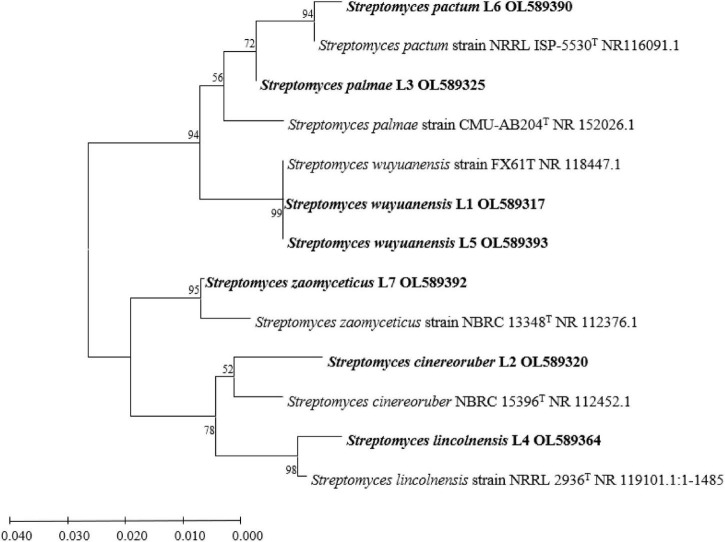
Phylogenetics tree based on16S rRNA gene sequence of isolates L1–L7 and *Streptomyces* type strains.

According to the full-length sequences of 16S rRNA genes, seven isolated actinobacterial strains in total were identified. All of these strains were assigned in *Streptomyces* genus with the identity of 16S rRNA gene > 97% as criteria. Based on the closest species in the sequences of 16S rRNA gene, the seven *Streptomyces* isolates were assigned to species level and named as *Streptomyces wuyuanensis* L1, *Streptomyces cinereoruber* L2, *Streptomyces palmae* L3, *Streptomyces lincolnensis* L4, *Streptomyces wuyuanensis* L5, *Streptomyces pactum* L6, and *Streptomyces zaomyceticus* L7 (Table 1). The phylogenetic relationship based on full sequences of 16S rRNA genes of all seven *Streptomyces* with corresponding type strains confirmed their taxonomy (Figure 1).

**TABLE 1:** Plant-promoting potentials of seven *Streptomyces* isolates.

Isolates	Most closely related species	GenBank accession number	Phosphate solubilization (SI)	Nitrogen fixation	ACC deaminase activity	IAA production (μg/mL)	Siderophore production
L1	*Streptomyces wuyuanensis*	OL589317	–	+	+	4.35 ± 0.07	20.55%
L2	*Streptomyces cinereoruber*	OL589320	1.41	+	+	3.89 ± 0.42	12.60%
L3	*Streptomyces palmae*	OL589325	–	+	–	3.55 ± 0.46	4.99%
L4	*Streptomyces lincolnensis*	OL589364	1.87	+	+	3.03 ± 0.31	2.36%
L5	*Streptomyces wuyuanensis*	OL589393	1.25	–	+	–	–
L6	*Streptomyces pactum*	OL589390	–	+	–	–	–
L7	*Streptomyces zaomyceticus*	OL589392	–	+	+	4.03 ± 0.51	6.04%

### Plant Growth Promoting Potential of Isolated *Streptomyces*

The plant growth promoting potential of seven isolated *Streptomyces* was characterized *in vitro* by determining qualitatively and/or quantitatively the ability of phosphate solubilization, nitrogen fixation, ACC deaminase, IAA, and iron carrier production (Table 1).

#### Phosphate Solubilization

Strains L2, L4, and L5 among these seven isolated strains were identified as phosphate solubilizing microbes according to their ability to solubilize inorganic phosphorus with the ratio between halo plus colony diameter and colony diameter of 1.41, 1.87, and 1.25, respectively. In addition, strain L4 has the ability to dissolve organic phosphorus (Supplementary Figure 2).

#### Nitrogen Fixation

Strains L1, L2, L3, L4, L6, and L7, except for strain L5, exhibited their nitrogen fixation ability judged by their apparent growth on nitrogen-free medium after at least three continuous transferring (Supplementary Figure 3).

#### Indole-3-Acetic Acid Production

**FIGURE 2:**
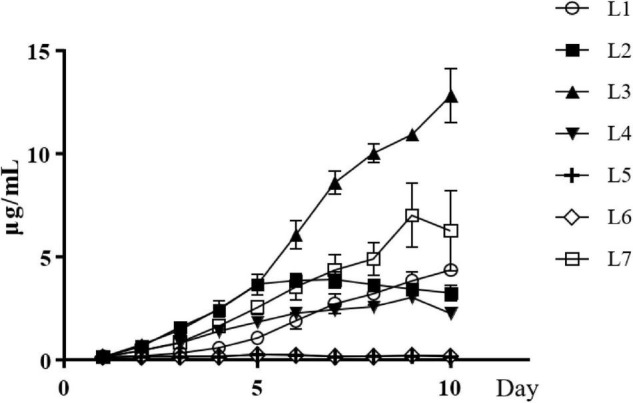
IAA production by seven *Streptomyces* isolates, *Streptomyces wuyuanensis* L1, *Streptomyces cinereoruber* L2, *Streptomyces palmae* L3, *Streptomyces lincolnensis* L4, and *Streptomyces zaomyceticus* L7.

The concentration of IAA in the culture of strain L1 kept increased after inoculation and reached to the highest value of 4.35 ± 0.07 μg/mL on the 10th day. The IAA concentration of strain L2 increased to the highest on the sixth day and stabilized with the value of 3.89 ± 0.42 μg/mL. Exceptionally, due to that strain L3 began to produce pigment on the fifth day and interfered the measurement of IAA, the highest value of IAA was observed on the fifth day as 3.55 ± 0.46 μg/mL. The production of IAA in strain L4 reached to the highest on the sixth day and decreased on the ninth day, with the highest value of 3.03 ± 0.31 μg/mL. Strain L7 also began to produce pigment on the seventh day, so the highest value of IAA, 4.03 ± 0.51 μg/mL, was observed on the fifth day. There was no IAA detected in strains L5 and L6 (Figure 2).

#### Siderophore Activity

The concentration of siderophore (iron carrier) in *Streptomyces* cultures was changed with incubation time when these strains were grown in iron carrier medium. The highest concentrations of iron carrier for each strain were 20.55% at 48 h for L1 and 12.6%, 4.99%, 2.36%, and 6.04% at 36 h for L2, L3, L4, and L7, respectively. There was no iron carrier activity in strains L5 and L6.

#### 1-Aminocyclopropane-1-Carboxylate Deaminase Activity

Strains L1, L2, L4, L5, and L7 were grown well after three continuous transferring on ADF medium supplemented with ACC as the sole nitrogen source, indicating their ACC deaminase activity for decomposing ACC into ACC butanoic acid and ammonia that could be utilized as direct nitrogen source by plants (Table 1).

### Root Growth Parameters

**FIGURE 3:**
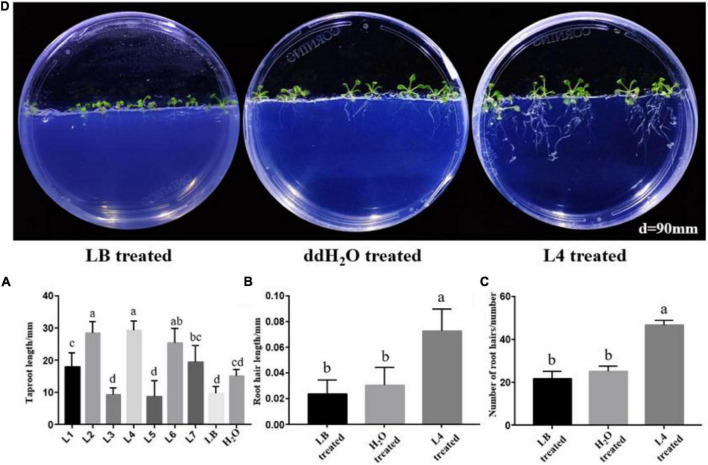
Root morphology influenced by the colonization of PGPR isolates. **(A)** The growth of *Arabidopsis thaliana* treated with *S*. *lincolnensis* L4; **(B)** changes in taproot length of *A*. *thaliana* after colonization by strain *Streptomyces wuyuanensis* L1, *Streptomyces cinereoruber* L2, *Streptomyces palmae* L3, *S*. *lincolnensis* L4, *Streptomyces wuyuanensis* L5, *Streptomyces pactum* L6, and *Streptomyces zaomyceticus* L7. It can be seen from the figure that L4 strain has the best effect on promoting the root length; **(C)** changes in root hair length after treatment by *S*. *lincolnensis* L4; **(D)** changes in number of root hairs per 3 mm taproot after treatment by *S*. *lincolnensis* L4.

**FIGURE 4:**
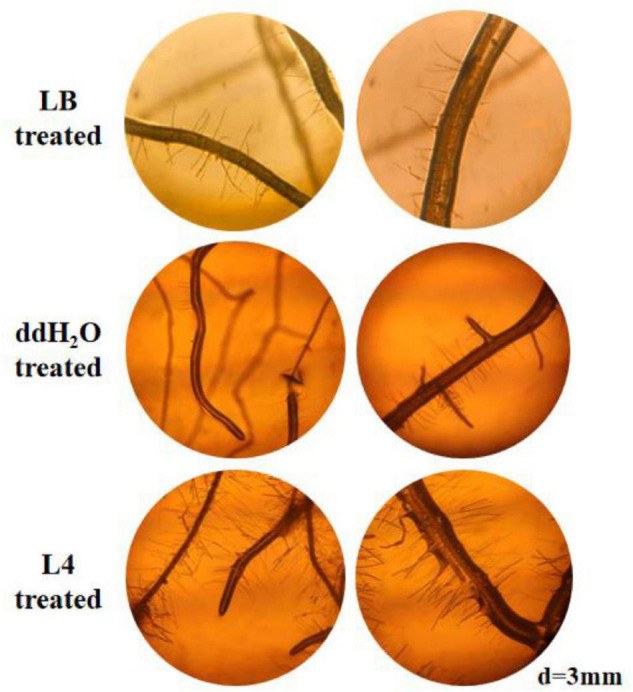
Microscopic observation of *A*. *thaliana* roots after treatment with *S*. *lincolnensis* L4.

As a primary indicator of root growth, the taproot lengths of *A*. *thaliana* treated by all seven *Streptomyces* isolates, LB medium and ddH_2_O, were measured. The taproot length treated by strains L1, L2, L4, L6, and L7 was increased in comparison with LB and ddH_2_O-treated control (Figures 3A,B). Strain *S*. *lincolnensis* L4 demonstrated the highest taproot length (32 mm) among all treatments. More than changes in taproots, the average length (Figure 3C), and number (Figures 3D, 4) of root hairs in *A*. *thaliana* treated by strain L4 were also promoted, which indicated the potential of strain L4 to change root morphogenesis. Thus, *S*. *lincolnensis* L4 was further investigated for its function in endophytic microbiota and its genomic trait.

### Effect of *Streptomyces lincolnensis* L4 on Root Endophytic Bacterial Community in *Arabidopsis thaliana*

From Illumina sequencing of 16S rRNA gene, 833578 sequences in total were obtained from three treatments including three root samples from L4-treated and three root samples from ddH_2_O-treated *A*. *thaliana*. After removing sequences from mitochondria and chloroplasts, an average of 14,295 reads per sample was generated, with a total of 382 bacterial OTUs, representing composition of endophytic bacterial community in *A*. *thaliana*. OTUs with abundance greater than 1% were defined as dominant.

**FIGURE 5:**
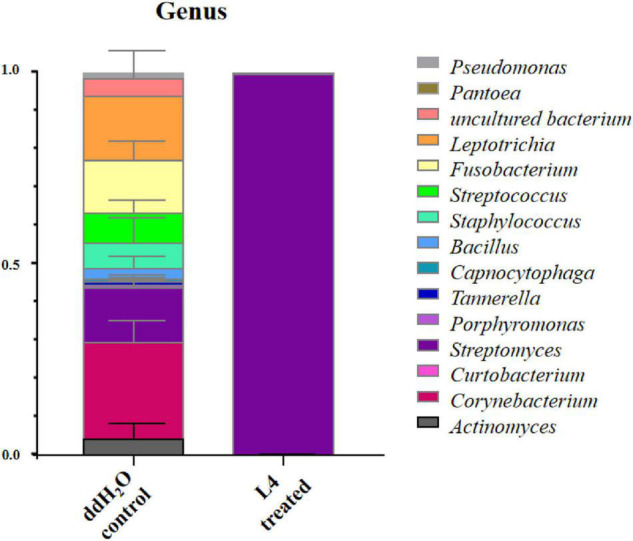
Endophytic bacterial community at genus level in the root of *A*. *thaliana* after treatment with *S*. *lincolnensis* L4.

The richness and diversity of endophytic bacterial communities were calculated. In comparison with ddH_2_O-treated control, the abundance of *Streptomyces* as dominant genus in the group treated with *S*. *lincolnensis* L4 was much higher than that in the ddH_2_O-treated control (Figure 5). These results indicated that *S*. *lincolnensis* L4 had been colonized into the root of *A*. *thaliana*. However, the bacterial alpha diversity, represented by Shannon index, was significantly reduced in *S*. *lincolnensis* L4 treatments (Supplementary Figure 4).

### Genome of *Streptomyces lincolnensis* L4

The assembled draft genome of *S*. *lincolnensis* L4 is 10.54 Mb in estimated length, comprising 82 scaffolds with an overall G+C content of 71.20%. A total of 9,334 protein-coding DNA sequences (CDS) and 100 predicted RNAs (92 tRNAs, 12 rRNAs, and 1 tmRNA) were identified from the draft genome. The genome sequence of *S*. *lincolnensis* L4 had been submitted NCBI GenBank with accession number PRJNA753994.

**FIGURE 6:**
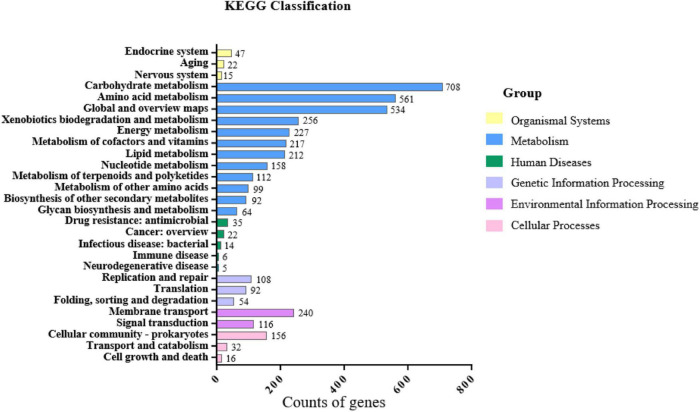
Genomic annotation of *S*. *lincolnensis* L4 using KEGG.

From the function annotation of KEGG, besides two major categories of central carbon metabolism “carbohydrate metabolism” (708 CDSs) and “amino acid metabolism” (561 CDSs), these characterized categories including “xenobiotics biodegradation and metabolism” (256 CDSs), “metabolism of terpenoids and polyketides” (112 CDSs), and “biosynthesis of other secondary metabolites” (92 CDSs) may be involved in plant growth promotion (Figure 6).

Specifically, genes encoding function of ACC deamination, iron transportation, and IAA synthesis were identified from genome in *S*. *lincolnensis* L4. The presence of gene encoding ACC deaminase (1-aminocyclopropane-1-carboxylate deaminase, EC 3.5.99.7) in L4 genome indicates its potential to promote plant growth by decreasing ethylene level resulting in extensive root growth (Sheehy et al., 1991). Furthermore, IucA/IucC family protein including aerobactin synthase (EC 6.3.2.39) (Lorenzo and Neilands, 1986) and N(2)-citryl-N(6)-acetyl-N(6)-hydroxylysine (EC 6.3.2.38) (Hickey and Cianciotto, 1997) functioned in siderophore biosynthesis was identified in L4 genome. In addition, several genes involved in IAA production were identified including genes encoding tryptophan 2-monooxygenase (EC 1.13.12.3), and aldehyde dehydrogenase AldA (EC 1.2.1.3), which play a role in the early stage of IAA synthesis (Krause et al., 2015; Mcclerklin et al., 2018). However, other encoding gene(s) involved in IAA synthesis was not annotated in the draft genome of *S*. *lincolnensis* L4 (Table 2).

**TABLE 2:** Genes involved in plant growth promotion in the genome of *Streptomyces lincolnensis* L4.

Function	Gene definition	Enzyme number
ACC deamination	1-Aminocyclopropane-1-carboxylate deaminase	EC 3.5.99.7
Iron transportation	IucA/IucC family siderophore biosynthesis protein	EC 6.3.2.-
	Aerobactin synthase	EC 6.3.2.39
	N(2)-citryl-N(6)-acetyl-N(6)-hydroxylysine	EC 6.3.2.38
IAA production	Tryptophan 2-monooxygenase	EC 1.13.12.3
	Aldehyde dehydrogenase AldA	EC 1.2.1.3

### Expression Changes of Root Development-Related Genes After Colonization of *Streptomyces lincolnensis* L4

**FIGURE 7:**
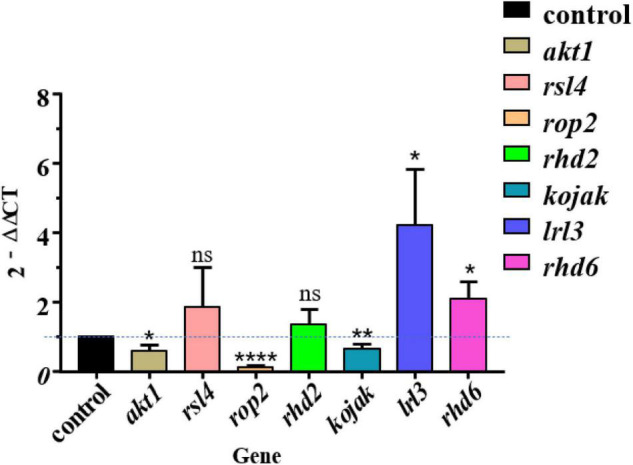
Quantitative PCR revealed the differential expression of genes involved in root development in *A*. *thaliana* after colonization of *S*. *lincolnensis* L4. Expression of four genes *lrl3* and *rhd6* were upregulated in the L4 treatment group compared with the control group; expression of three genes *akt1*, *kojak*, and *rop2* were decreased; *rsl4* and *rhd2* have no significant difference compared with the control group. The “2^–ΔΔ*C*T^” method was used for calculating the expression change of target genes with 18S rRNA gene as housekeeping gene, in which 2^–ΔΔ*C*T^> 1 or <1 represents the upregulation or downregulation of target genes. The significances were presented by *P < 0.05; **P < 0.01; ****P < 0.0001.

To further explore the role of *S*. *lincolnensis* L4 in the process of root hair elongation of *A*. *thaliana*, we selected seven genes related to root hair growth and development (*akt1*, *kojak*, *rop2*, *lrl3*, *rsl4*, *rhd6*, and *rhd2*) and quantified their expression in the root of L4-treated *A*. *thaliana* by performing real-time quantitative PCR with primers reported in the previous study (Forman and Torres, 2002; Sieberer et al., 2005; Dias and Sá-Correia, 2013; Nakamura et al., 2018; Wang et al., 2019; Supplementary Table 1). Expression of four genes *lrl3* and *rhd6* was upregulated in treatment group; expression of three genes *akt1*, *kojak*, and *rop2* was decreased; *rsl4* and *rhd2* have no significant difference compared with the control group (Figure 7). These upregulated genes implied their function in root development induced by the colonization of *S*. *lincolnensis* L4, in which gene *lrl*3 encodes AtLRL3 that mediate the development of root hair cells in *Arabidopsis* by acting downstream from the regulatory complexes that specify pattern formation in the epidermis (Karas et al., 2009), and gene *rhd*6 controls the number of root hairs (Menand et al., 2007).

On the contrary, expression of Rop2 GTPase-encoding gene (*rop2*) was downregulated by the inoculation of *S*. *lincolnensis* L4 at late stage of root development in our treatment, probably due to that it is a positive regulator of root hair initiation (Jones et al., 2002). Similarly, both gene *akt*1 and gene *kojak* were downregulated due to the inoculation of strain L4. Gene *akt*1 is an important gene for K^+^ transport in roots (Wang et al., 2016). Gene *kojak* encoded KOJAK/AtCSLD3 (KOJAK encodes a cellulose synthase-like protein, AtCSLD3) function in the biosynthesis of β-glucan-containing polysaccharides that are required during root hair elongation (Favery et al., 2001). This may be also because they function in the earliest stage but the analysis was conducted on the late stage of root hair development. Gene *rhd2* facilitates Ca^2+^ influx for growth (Foreman et al., 2003) and *rsl4* encodes RSL4 protein involved in signaling, secretion, and cell wall modification (Vijayakumar et al., 2016). Expression of the two genes was significant, which may be related to the stabilization of gene expression in the later stage of root growth and development.

## Discussion

The plant growth promotion could be the result of the beneficial functions of applied PGPR isolates, such as plant growth hormone production, nitrogen fixation, and P solubilization, and these microorganisms with the function of promoting plant growth and development will be optimal substitute of chemical fertilizers (Hassen et al., 2018). In this study, a plant growth-promoting bacterium, *S*. *lincolnensis* L4, was isolated from the rhizosphere soil of *A*. *annua*. In the aseptic seedling experiment, *S*. *lincolnensis* L4 had a positive effect on the root growth and development of *Arabidopsis*. Compared with control, root length, number of root hairs, and length of root hair were improved after the colonization of L4 in roots. The results of real-time quantitative PCR showed that the expression of genes involved in root development in *Arabidopsis* was regulated by the inoculation of L4 culture which verified the interaction between *S*. *lincolnensis* L4 and host plant. The analysis of community composition and structure of endophytic bacteria showed that the relative abundance of *Streptomyces* reached more than 99.8% in the root of *A*. *thaliana* treated by *S*. *lincolnensis* L4, which had become the dominant endophytes compared with other genera, indicating that *S*. *lincolnensis* L4 as PGPR bacteria had actual interaction with *A*. *thaliana* by colonization in its root. These results, combined with IAA production, ACC deamination, and iron carrier production, revealed that *S*. *lincolnensis* L4 harbors the ability to stimulate plant root growth by tuning its root morphogenesis. On the other hand, the domination of *Streptomyces* spp. in roots reduced the alpha diversity, which may have risks to break the ecological balance of endophytic community even though negative effect to root growth and development was not observed in this study (Sagita et al., 2021). The changes in endophytic microbial diversity which cause by alien microbial inoculation should be considered during PGPR strategic design, which could be optimized in the proper inoculating dosage or synthetic consortium.

It has been well studied that siderophore production and IAA synthesis are related to efficient nutrient uptake and significant enhancement of the root system, respectively (Patten and Glick, 2002; Etesami et al., 2015; Abbamondi et al., 2016). Siderophore-producing bacteria indirectly promoted plant growth by preventing the proliferation of pathogens by decreasing the amount of available iron (Olanrewaju et al., 2017; Compant et al., 2019). The majority of the isolated bacteria in this study, including *S*. *lincolnensis* L4, were able to synthesize IAA, indicating its potential of PGPR trait similar to the previous studies (Spaepen and Vanderleyden, 2011). However, the observed concentration of IAA changed probably due to the various pathways for biosynthesis of IAA in different species (Majeed et al., 2015). High levels of IAA promote the formation of lateral roots (Gupta and Pandey, 2019) and increase root length (Olanrewaju et al., 2017). Even though the IAA synthesis pathway was not completely annotated in the draft genome of strain L4, the apparent IAA production and promoted root development demonstrated its function to regulated root growth *via* IAA accumulation. On the other hand, ACC deaminase activity that produces certain amount of α-ketobutyrate was observed in *S*. *lincolnensis* L4. As a product of ACC deamination, ammonia could be utilized by plant as direct nitrogen, which has been proved in other PGPR strains (Penrose and Glick, 2003; Checcucci et al., 2017; Jaemsaeng et al., 2018). These above-described PGPR traits in *S*. *lincolnensis* L4 were further verified in genomic analysis and quantitative assay of root growth-related genes in *Arabidopsis*, which conclusively implied its role in plant growth promotion through regulating root development.

## Conclusion (Final Remark)

From the aspects of microbial physiology, genomic analysis, endophytic bacterial community, root phenotype, and gene expression of host plant, we have demonstrated that isolated *Streptomyces* strains harbor various traits including N_2_ fixation, P-solubilizing, siderophore activity, ACC deaminase activity, and IAA-producing bacteria which present among the natural population. Through these characteristics, strain L4 is specifically capable of regulating the root length and root hair number of *Arabidopsis* which was verified by the expression of gene related to root development. Thus, this *Streptomyces* strain offers potential in field applications as root growth-promoting agents. Further studies should be focused on the detailed molecular and functional characterization of these PGPR for unraveling the mechanism of root development driven by associated microbes.

Plant growth-promoting rhizobacteria have been proved on their ability of nutrient acquisition, phosphorus solubilization and phytohormones synthesis in crops. It has been considered as a feasible alternative to chemical approaches (Olanrewaju et al., 2017). These metabolic potentials of *Streptomyces* in plant growth promotion have received increasing attentions (Olanrewaju and Babalola, 2019). We believe that these microorganisms with the function of promoting plant growth and development will be optimal substitute of chemical fertilizers, and also ensure the sustainable development of agriculture.

## Data Availability Statement

The datasets generated for this study can be found in the NCBI under accession numbers PRJNA753994 and PRJNA761252. The genome of Streptomyces lincolnensis L4 is available at: https://www.ncbi.nlm.nih.gov/bioproject/PRJNA753994. The community data are available at: https://www.ncbi.nlm.nih.gov/bioproject/PRJNA761252.

## Author Contributions

WF: conceptualization, methodology, formal analysis, investigation, data curation, and writing—original draft. YP and YS: conceptualization, validation, resources, and investigation. JC: writing—review and editing and visualization. DG and YL: writing—reviewing and editing. GH: writing—reviewing and editing and supervision. DH: writing—reviewing and editing, supervision, project administration, and funding acquisition. All authors contributed to the article and approved the submitted version.

## Conflict of Interest

The authors declare that the research was conducted in the absence of any commercial or financial relationships that could be construed as a potential conflict of interest.

## Publisher’s Note

All claims expressed in this article are solely those of the authors and do not necessarily represent those of their affiliated organizations, or those of the publisher, the editors and the reviewers. Any product that may be evaluated in this article, or claim that may be made by its manufacturer, is not guaranteed or endorsed by the publisher.
