# Supplementary material for: Root Morphogenesis of Arabidopsis thaliana Tuned by Plant Growth-Promoting Streptomyces Isolated From Root-Associated Soil of Artemisia annua
Source: Front Plant Sci. 2022 Jan 10;12:802737. doi: 10.3389/fpls.2021.802737 (PMC8786036; doi:10.3389/fpls.2021.802737)
Supplement: Supplementary file 1 [file Table_1.docx]

**Table S1.** Primer sequences for quantitative PCR of root development related genes in *Arabidopsis thaliana*.

| Primers | Sequence (5’-3’) |
| --- | --- |
| 18S rRNA-F | CGGCTACCACATCCAAGGAA |
| 18S rRNA-R | GCTGGAATTACCGCGGCT |
| ROP2 QF | GGATTGTGGGATACTGCTGGTC |
| ROP2 QR | CCAACAAGGATAATGGGAACA |
| KOJAK QF | GATGAGCAGACCGTGGAGAC |
| KOJAK QR | TAGCCAAGATAAGGCGAACC |
| AKT1 QF | TTGGACGCCGAGGGAT |
| AKT1 QR | CAGGACGGATGTTGGGTT |
| RHD2 QF | AGGTTGGGTCGTGTTGTCC |
| RHD2 QR | ATGCCAGTAGCTCTTTGGTTG |
| LRL3 QF | TCAGTTGGTCCACGCCTCA |
| LRL3 QR | CATCAGTTTCGCCACTCTTTGTT |
| RSL4 QF | GGACAGCCACTGATCCTCAA |
| RSL4 QR | CACGTAATGGACCGCTTCT |
| RHD6 QF | GGGCGGCTTCTCCTTCT |
| RHD6 QR | GTTTGTTTCCAGCGGATTTAG |
