# Supplementary figures and images for: Root Morphogenesis of Arabidopsis thaliana Tuned by Plant Growth-Promoting Streptomyces Isolated From Root-Associated Soil of Artemisia annua
Source: Front Plant Sci. 2022 Jan 10;12:802737. doi: 10.3389/fpls.2021.802737 (PMC8786036; doi:10.3389/fpls.2021.802737)

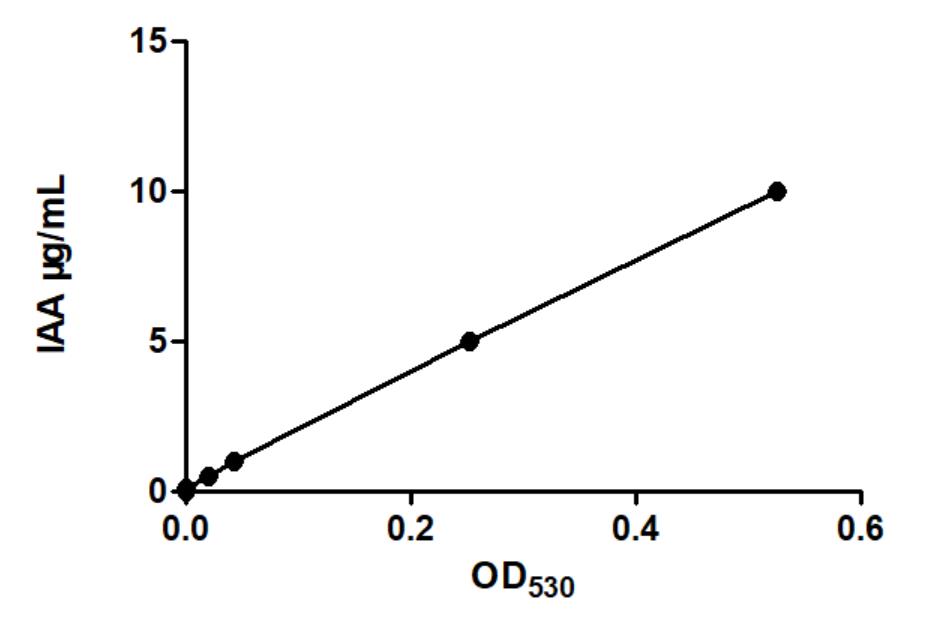

Supplement: Supplementary file 2 [file Image_1.JPEG]

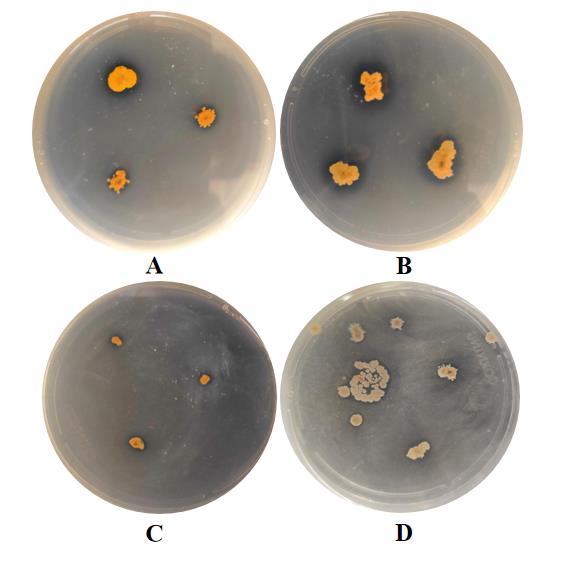

Supplement: Supplementary file 3 [file Image_2.JPEG]

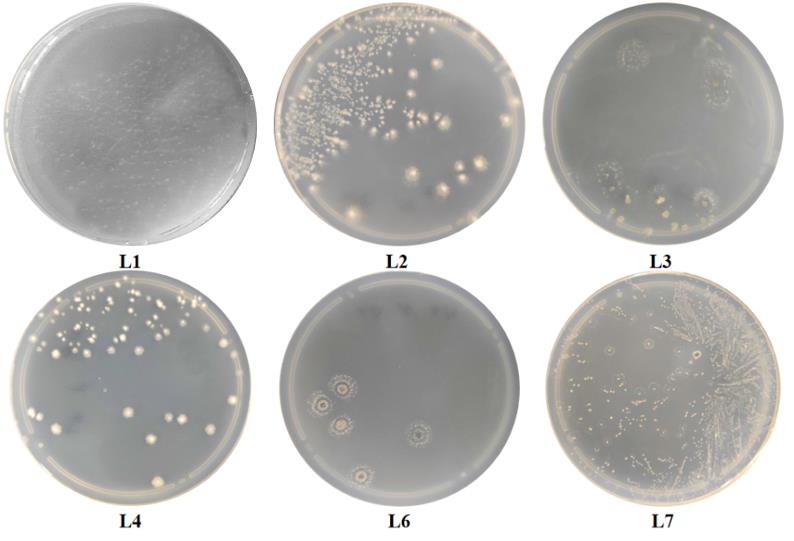

Supplement: Supplementary file 4 [file Image_3.JPEG]

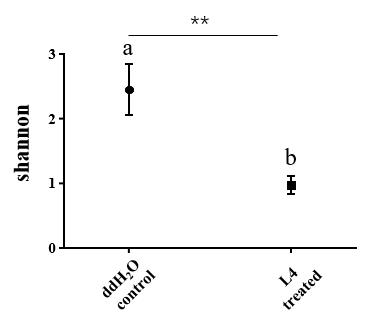

Supplement: Supplementary file 5 [file Image_4.JPEG]
